# A second occurrence of intravitreal injection-associated endophthalmitis 2 years apart

**DOI:** 10.1016/j.ajoc.2026.102525

**Published:** 2026-01-30

**Authors:** Landon J. Rohowetz, Harry W. Flynn, Justin H. Townsend

**Affiliations:** Department of Ophthalmology, Bascom Palmer Eye Institute, Miami, FL, USA

**Keywords:** Endophthalmitis, Anti-VEGF, Intravitreal injection

## Abstract

**Purpose:**

This case report describes a patient who developed a second episode of endophthalmitis in the same eye following intravitreal injections administered 2 years apart.

**Observations:**

An 83-year-old pseudophakic male with a history of neovascular age-related macular degeneration and diabetes mellitus complained of decreased visual acuity and pain in the left eye 1 day after receiving intravitreal aflibercept 2 mg. Best-corrected visual acuity (BCVA) was light perception. The clinical examination revealed signs of endophthalmitis including fibrin and hypopyon while B-scan ultrasonography demonstrated vitreous opacities and membranes. The patient underwent vitreous tap and injection with intravitreal vancomycin, ceftazidime, and dexamethasone. Pars plana vitrectomy with posterior hyaloid detachment was performed the following day due to a relative lack of clinical improvement. Vitreous cultures ultimately demonstrated no growth and BCVA improved to baseline. Anti-VEGF therapy was resumed 3 months after the initial diagnosis of endophthalmitis. Two years later the patient presented with evidence of a second episode of endophthalmitis 4 days after receiving aflibercept 8 mg. Vitreous tap and injection was again performed and cultures revealed growth of *Staphyloccoccus epidermidis*. The patient demonstrated marked improvement with no evidence of residual inflammation at 2 weeks. Best-corrected visual acuity was 20/50 at 7-month follow-up examination.

**Conclusion and importance:**

Endophthalmitis is a rare complication of intravitreal injection. The current report describes a patient who developed a second occurrence of endophthalmitis associated with an intravitreal injection 2 years later.

## 1 Introduction

Intravitreal anti-vascular endothelial growth factor (VEGF) therapy is the mainstay of treatment for many retinal vascular diseases.^1^ Endophthalmitis, an infection involving the vitreous and/or aqueous humor, is a rare complication of intravitreal injection.^2^, ^3^, ^4^ Management generally consists of vitreous tap with administration of intravitreal antibiotics. Pars plana vitrectomy is typically reserved for severe cases and those nonresponsive to conservative management.^5^ The prognosis is frequently guarded and dependent upon the causative organism.^6^ In this report, we present a patient with a second episode of endophthalmitis following intravitreal injections administered 2 years apart.

## 2 Case report

**Fig. 1:**
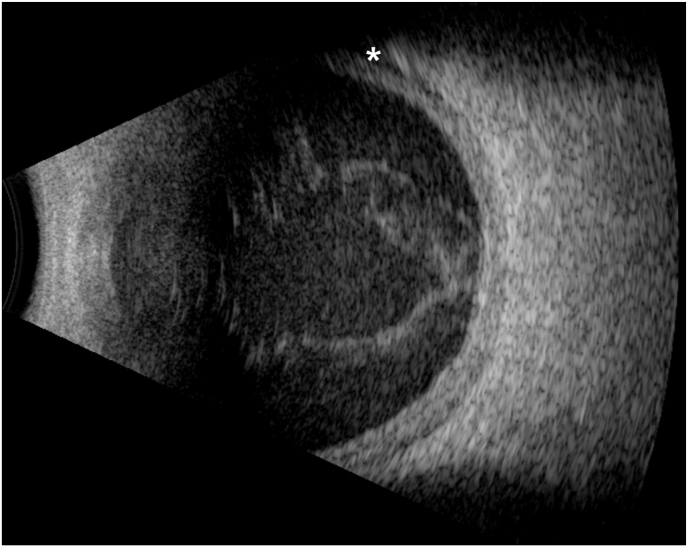
**B-scan ultrasonography at first episode** B-scan ultrasonography of an 83-year-old male with suspected post-intravitreal injection-associated endophthalmitis demonstrating vitreous opacities and membrane formation (arrows) with a shallow choroidal detachment (asterisk).

An 83-year-old male presented to the eye emergency room complaining of pain and decreased vision in the left eye. The patient had a history of neovascular age-related macular degeneration and had received intravitreal aflibercept 2 mg/0.05 mL the day prior, at which time his best-corrected visual acuity (BCVA) was 20/40. The injection was administered through the inferotemporal pars plana without the use of lidocaine gel. He had been receiving intravitreal aflibercept 2 mg at regular 8-week intervals. Past medical history was remarkable for diabetes mellitus with a hemoglobin A1c of 6.3%. He had undergone cataract surgery with phacoemulsification 6 years prior. There was no history of eyelid abnormalities including ectropion, entropion, blepharitis, or meibomian gland dysfunction. Upon presentation to the emergency department, BCVA was light perception and intraocular pressure was 28 mmHg. Anterior segment examination demonstrated conjunctival injection, microcystic corneal edema, hypopyon, fibrin, pupillary membrane formation, and posterior synechiae with a posterior chamber intraocular lens. There was no view to the posterior segment. B-scan ultrasonography demonstrated vitreous opacities and membrane formation with a shallow choroidal detachment (Fig. 1). The patient underwent vitreous tap and injection with intravitreal vancomycin 1 mg/0.1 mL, ceftazidime 2.25 mg/0.1 mL, and dexamethasone 0.4 mg/0.1 mL. The following day, visual acuity remained light perception and the examination was unchanged. Pars plana vitrectomy with posterior hyaloid detachment was pursued after discussion of the risks and benefits. Intraoperative examination demonstrated dense vitritis and membranes with purulent material on the preretinal surface and scattered diffuse intraretinal hemorrhages. An undiluted vitreous sample was obtained for culture and intravitreal vancomycin, ceftazidime, and dexamethasone were again administered. By postoperative week 2, BCVA had improved to 20/70. At postoperative month 3, evidence of inflammation was resolved. Gram stain was unremarkable and cultures demonstrated no growth. Intravitreal anti-VEGF therapy was resumed 3 months after the initial diagnosis of endophthalmitis.

**Fig. 2:**
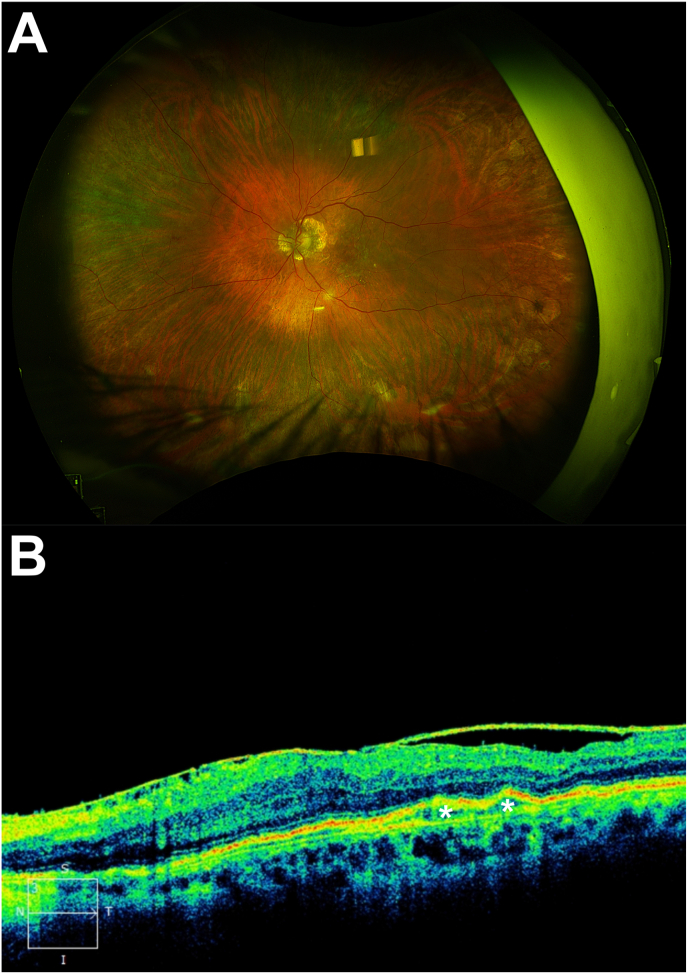
**Fundus photography and optical coherence tomography 2 weeks after second presentation** Fundus photography (A) of an 85-year-old male 2 weeks after initial vitreous tap and injection of ceftazidime, vancomycin, and dexamethasone for intravitreal injection-associated endophthalmitis demonstrating clear vitreous with no evidence of endophthalmitis or intraocular inflammation. Optical coherence tomography (B) demonstrates stable pigment epithelial detachments (asterisks) and an epiretinal membrane (arrows) without evidence of vitreous cells.

Two years later, the patient presented to the eye emergency department complaining of eye pain and blurred vision in the same left eye 4 days after receiving aflibercept 8 mg/0.07 mL. The injection was again administered through the inferotemporal pars plana without the use of lidocaine gel. He had received a total of 22 anti-VEGF injections since his diagnosis of endophthalmitis and had been receiving intravitreal aflibercept 8 mg at regular 5-week intervals. Best-corrected visual acuity was 20/400 from 20/50 and intraocular pressure was 10 mmHg. Examination was consistent with endophthalmitis, demonstrating keratic precipitates, 4+ cell and flare, hypopyon, fibrin, and vitritis. B-scan ultrasonography demonstrated dense vitreous membranes. Vitreous tap and injection was performed with intravitreal vancomycin 1 mg/0.1 mL, ceftazidime 2.25 mg/0.1 mL, and dexamethasone 0.4 mg/0.1 mL. Visual acuity was hand motions with slight improvement of inflammation on examination 3 days later. Gram stain revealed growth of gram positive cocci so intravitreal vancomycin and dexamethasone were again administered at that time. Four days later, BCVA improved to 20/150. There was no anterior or posterior segment inflammation and the hypopyon resolved. Vitreous culture demonstrated heavy growth of *Staphylococcus epidermidis*. Examination and optical coherence tomography (OCT) were improved 1 week later without evidence of persistent infection or inflammation (Fig. 2). Seven months after the second occurrence of endophthalmitis, BCVA in the left eye was 20/50 and OCT demonstrated stable intraretinal cystic spaces in the macula. The patient has been monitored monthly but has not experienced recurrent exudation and as such has not received further anti-VEGF treatment.

## 3 Discussion

Endophthalmitis is a rare complication of intravitreal anti-VEGF injection with an incidence between 0.013% and 0.061%.^2^^,^^7^ The most common causative organisms are coagulase-negative *Staphylococci* and *Streptococcus* species.^7^ Infections with *Streptococcus* species are associated with worse visual outcomes compared to infections caused by coagulase-negative *Staphylocci* and culture negative cases.^8^ The current patient developed culture-negative endophthalmitis and *Staphylococcus epidermidis*-associated endophthalmitis after receiving intravitreal injections 2 years apart.

Risk factors for the development of endophthalmitis in the described patient included older age and diabetes mellitus. The patient had no history of functional eyelid abnormalities such as ectropion and lidocaine gel was not used during either injection. Older age is a risk factor for endophthalmitis potentially as a result of reduced immune system function and ocular surface abnormalities including decreased tear production.^9^ Likewise, diabetes mellitus is associated with impaired immune function, increased bacterial colonization, and poor wound healing.^10^^,^^11^ A prior history of endophthalmitis may also indicate the presence of a potential vulnerability in the defense mechanisms needed to prevent infection and a higher aggregate number of intravitreal injections may predispose patients to the development of endophthalmitis as demonstrated by prior authors.^12^

Although rare, a second episode of endophthalmitis in the same eye has been reported, most commonly after cataract surgery. However, most cases have occurred within weeks of the first diagnosis and therefore may represent an uncovering or exacerbation of an incompletely treated infection.^13^ These instances are most frequently seen in patients with postcataract surgery enterococcal endophthalmitis due to dormancy of the bacteria within the capsular bag.^12^ In 2021, Shields and colleagues reported 8 of 535 (1.5%) patients with a history of endophthalmitis who developed a second episode of endophthalmitis following a period of complete resolution of inflammation. Intravitreal injection was the cause of both endophthalmitis episodes in 3 of these patients. Coagulase-negative *Staphylococcus* was the causative organism in 2 of the 3 patients and BCVA returned to near baseline in all 3 patients. Two patients in the aforementioned study had undergone prior vitrectomy. The impact of vitrectomy on endophthalmitis risk is incompletely understood although theoretical considerations suggest that these eyes may be more susceptible to infection. Vitrectomy increases fluid convection and may allow organisms more direct access to posterior segment structures in addition to altering immunologic defense mechanisms. On the other hand, the risk of endophthalmitis may be lower in previously-vitrectomized eyes due to absence of vitreous which may act as a colonization medium for microorganisms and the faster clearance of foreign material in previously vitrectomized eyes as demonstrated by prior pharmacokinetic data.^14^^,^^15^ Regardless, this case highlights the potential for recurrent endophthalmitis in vitrectomized eyes, emphasizing the need for continued vigilance in patients with a history of endophthalmitis and altered vitreous anatomy, even though the impact of prior vitrectomy remains unclear.

## 4 Conclusions

Endophthalmitis is an infrequent complication of intravitreal injection. In this case report, we describe a patient with a second episode of endophthalmitis following intravitreal injections administered 2 years apart. This case highlights the importance of postprocedural surveillance, particularly in patients with known risk factors or a prior history of endophthalmitis.

## CRediT authorship contribution statement

**Landon J. Rohowetz:** Writing – review & editing, Writing – original draft, Data curation. **Harry W. Flynn:** Writing – review & editing, Supervision, Data curation, Conceptualization. **Justin H. Townsend:** Writing – review & editing, Writing – original draft, Supervision, Project administration, Investigation, Data curation, Conceptualization.

## Patient consent

The patient consented to publication of the case in writing.

## Authorship

All authors attest that they meet the current ICMJE criteria for Authorship.

## Funding

Research to Prevent Blindness-Unrestricted Grant to BPEI (GR004596-1; New York, NY). The funding sources had no role in study design, data collection, analysis and interpretation of data, writing of the report, or in the decision to submit the article for publication.

## Declaration of competing interest

The authors declare the following financial interests/personal relationships which may be considered as potential competing interests: JHT is a consultant for Bausch & Lomb and Genentech. The following authors have no financial disclosures: LJR, HWF.
